# Study of the Performance of DSSS UAC System Depending on the System Bandwidth and the Spreading Sequence

**DOI:** 10.3390/s21072484

**Published:** 2021-04-02

**Authors:** Iwona Kochanska, Roman Salamon, Jan H. Schmidt, Aleksander M. Schmidt

**Affiliations:** Faculty of Electronics, Telecommunications and Informatics, Gdańsk University of Technology, G. Narutowicza 11/12, 80-233 Gdańsk, Poland; roman.salamon@pg.edu.pl (R.S.); jan.schmidt@pg.edu.pl (J.H.S.); aleksander.schmidt@pg.edu.pl (A.M.S.)

**Keywords:** underwater acoustic communications, UAC, direct sequence spread-spectrum, DSSS, m-sequences, Kasami codes, Gold codes, multipath propagation

## Abstract

A signal transmitted in an Underwater Acoustic Communication (UAC) system operating in a shallow-water channel suffers from strong time dispersion due to multipath propagation. This causes the Inter-Symbol Interference (ISI) observed in the received signal, which significantly limits the communication system’s reliability and transmission rate. In such propagation conditions, the Direct-Sequence Spread Spectrum (DSSS) method is one of the solutions that make reliable data transmission possible. In systems with one-to-one communication, it ensures communication with a satisfactory Bit Error Rate (BER). Additionally, it makes it possible to implement the Code-Division Multiple Access (CDMA) protocol in underwater acoustic networks. This paper presents the results of simulation and experimental communication tests on a DSSS-based UAC system using three types of spreading sequence, namely m-sequences, Kasami codes and Gold codes, and occupying different bandwidths from 1 kHz to 8 kHz around a carrier frequency equal to 30 kHz. The UAC channel was simulated by impulse responses calculated by the virtual sources method and the UAC chanel models available in the Watermark simulator. The experimental tests were conducted in a model pool. Based on the obtained results, a transmission rate was estimated, which is possible to achieve in strong multipath propagation conditions, assuming reliability expressed as BER less than 0.001.

## 1. Introduction

Underwater acoustics communication systems are used in both civil and military applications, including control of Autonomous Underwater Vehicles (AUVs) and military objects on the seabed, instrument monitoring, pollution control, climate recording, prediction of natural disturbances, search and survey missions, and the study of marine life [1]. If the system operates in shallow water, the transmitted signal suffers from strong time dispersion due to multipath propagation. The received signal consists of reflections from the sea-bottom and the water’s surface and other objects present in the water [2]. Multipath propagation additionally includes strong refraction caused by a significant change in sound velocity as a function of depth. Both multipath propagation and refraction produce time dispersion of the transmitted signal, which causes Inter-Symbol Interference (ISI), the consequence of which is frequency-selective fading observed in the received signal spectrum. Moreover, if the UAC transmitter or receiver, or objects reflecting the signal, remain in motion, correct information detection possibilities are significantly limited due to the Doppler effect causing signal spectrum distortions.

Spread-Spectrum (SS) techniques are used in modern wireless communication systems due to their interference suppression benefits, energy density reduction, fine time resolution, and enabling of multiple access. The many features of pseudo-random signal processing techniques that are important for spread spectrum communication include the ability to cope with multipath propagation, resistance to interference, and the potential of sharing spectrum resources with other users. Spread-spectrum systems have a low probability of intercept (LPI). Transmitter–receiver pairs using independent random carriers can operate in the same bandwidth, with minimal co-channel interference. Moreover, SS systems have cryptographic capabilities, where the data modulation cannot be distinguished from the carrier modulation, and the carrier modulation is effectively random to an unwanted observer [3].

One of the SS techniques is the Direct-Sequence Spread Spectrum (DSSS) method. The DSSS signal occupies a much wider bandwidth than the minimum bandwidth needed to send the information. The spreading is implemented as a multiplication of the sequence of information bits with a Pseudo-Noise (PN) sequence independent of the data. At the receiver, recovery of original data is performed by correlation of the received spread signal with a replica of the spreading sequence [4].

Spread spectrum techniques are attractive for designers of UAC systems operating in shallow-water channels [5,6]. The DSSS technique is used in systems with one-to-one communication due to its resistance to interference caused by multi-path propagation. In addition, it is useful in underwater acoustic networks, making the implementation of Direct-Sequence (DS) Code-Division Multiple Access (CDMA) possible.

A PN-sequence used in a DSSS system as a spreading sequence should have good autocorrelation properties to achieve matched filtration benefits at the receiver. Hence, one-to-one communication systems often use m-sequences. However, in CDMA systems, the spreading sequences should have both good autocorrelation and cross-correlation properties. In such a system, each communication participant uses a unique PN-sequence when sending and receiving messages. The transmitted signals should not interfere with each other; therefore the PN-sequences should be mutually orthogonal, and thus its cross-correlation should be equal to zero. There are very few m-sequences of any given length with good cross-correlation properties. As a consequence, when numerous PN-sequences are needed, one needs to use Gold sequence sets or Kasami sequence sets, which have good cross-correlation properties and good autocorrelation properties [7].

There are numerous reported UAC systems using the DSSS technique. For example, in [8], DSSS signaling was used to increase the Signal-to-Noise Ratio (SNR) per data symbol and resolve multipath components for a single-user, short-range application. The system utilized 10 kHz of bandwidth to transmit the information with a rate equal to 625 bps at a distance of 1 km in shallow water. In [9] an improvement in the reliability of data transmission from BER of less than $10^{-1}$ to BER less than $10^{-2}$ in a system with QPSK modulation was achieved after implementing the DSSS technique. The Mediterranean Sea experimental results presented in [10] include data transmission tests at a distance of 45 nautical miles, using Gold sequences of rank 6 and achieving a data rate equal to 35.7 bps. An experiment at a distance of 20 km is also described, where using Kasami sequences of rank 6 made it possible to transmit data with a rate equal to 24 bps. In [11], the authors proposed a UAC modem, in which two Gold codes of length 2047 were used to spread differentially coded data bits. The bit rate of the system was 100 bps, and the carrier frequency was set to 12 kHz. The proposed modem was tested in the Baltic sea at an approximate range of about 3000 m. The authors obtained errorless transmission of blocks of 200 data bits. A DS CDMA underwater acoustic communication network is described in [12]. Communication between four users was demonstrated. Simulation tests showed that it is possible to achieve reliable data transmission at a rate of up to 2.5 kbps per user at a distance of 2 km, using the Quadrature Phase-Shift Keying (QPSK) modulation scheme and Kasami spreading codes of lengths 15, 63, and 255. The carrier frequency was equal to 33 kHz. In [13] the results of the stationary communication experiment are described. The data has been transmitted with a rate equal to about 0.5 bps at a distance of up to 8 km, using the DSSS signal of bandwidth equal to 2 kHz over carrier frequency equal to 12 kHz. M-sequence of rank 8 has been used as a spreading sequence. The article [14] describes a UAC modem performing data transmission using several physical layer configurations. One of them is the DSSS transmission mode, in which every QPSK symbol is spread by a portion of an m-sequence of rank 13. The band of the signal is equal to 8–16 kHz. During the experimental tests at a distance of 1.5 km, a data transmission rate of 100 bps was achieved. The Autonomous Underwater Vehicle, HUGIN, by Kongsberg Maritime, uses a commercial DSSS-based UAC modem, which operates at a horizontal range of up to 4 km in shallow water with a data transmission rate equal to 50 bps, but the manufacturer reserves that this value depends on the sound profile and acoustic noise [15].

The DSSS-based UAC systems described in the literature use specific PN sequences and fixed transmission bandwidth. To the best of the authors’ knowledge, there are no publications presenting an analysis of an underwater DSSS system’s performance depending on its bandwidth or the PN sequence used. This paper presents the results of simulation and experimental communication tests of a DSSS-based UAC system using three types of spreading sequence, namely m-sequences, Kasami codes, and Gold codes, and occupying different bandwidths from 1 kHz to 8 kHz around a center frequency equal to 30 kHz. The simulation tests of such a system using an Additive White Gaussian Noise (AWGN) channel model, Rician fading channel model, and a replay channel modeled by impulse responses measured during the inland water experiment were previously described in [16]. In this paper, the UAC channel was modeled by impulse responses calculated by the virtual sources method and by the Watermark simulator. The experimental tests were conducted in the model pool. During the tests, the bit error rate (BER) of data transmission was estimated. The goal of this simulation and experimental research was to estimate the transmission rate which is possible to achieve in strong multipath propagation conditions, assuming a reliability determined by the BER of lower than 0.001. This is an acceptable error rate for underwater telemetry systems requiring low data rates. A BER less than 0.01 is also considered as sufficient, for example, for poor-quality systems of speech signal transmission between divers and to a surface station [17].

## 2. Structure of DSSS Signals

**Figure 1:**
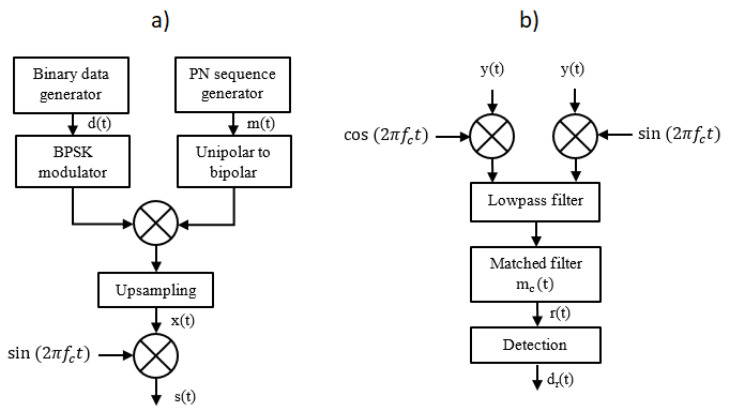
Modulator (**a**) and demodulator (**b**) block schemes of the DSSS UAC system.

The process of communication signal generation in the transmitter of the DSSS UAC systems is as follows. Figure 1a shows a block diagram of a DSSS modulator. The input data stream d(t) is formed into real-value Binary Phase Shift Keying (BPSK) constellation symbols from a set {−1; 1}. Next, the symbols are combined with PN sequence m(t) in such a way that a single PN sequence is multiplied by a single BPSK symbol. The product is upsampled by a factor of $R=f_{s}/B$, where $f_{s}$ is the sampling frequency equal to 200 kHz, and *B* denotes the system bandwidth. Such a prepared digital signal x(t) modulates the phase of the carrier wave of frequency $f_{c}$ equal to 30 kHz. The choice of carrier frequency was determined by the configuration of the laboratory model of the UAC system, which was used to perform experimental tests in the measurement pool—in particular, by the operating bandwidth of the ultrasonic transducers.

Three types of PN code were applied in such a configured DSSS system: m-sequences, Kasami codes, and Gold codes, all of rank 8, so their length was equal to 255 samples. Test signals occupying different frequency bands were prepared using each type of PN sequence. The values of bandwidth *B* are shown in Table 1. The corresponding upsampling factor *R*, the length of a single modulation symbol $N_{s}$, and its duration $T_{s}$ are also presented for each value of *B*. Sampling frequency $f_{s}$ is assumed to be equal to 200 kHz. The symbol duration $T_{s}$ determines the data transmission rate, calculated as a number of DSSS symbols per second (each of the DSSS symbols represents a single bit of information). The symbols are organized in data frames, whose duration is equal to 6 s. The number of symbols *K* in a single data frame is also shown in Table 1.

**Figure 2:**
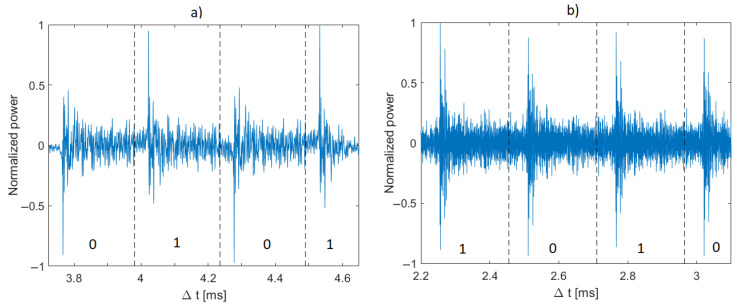
Real parts of exemplary signals at the output of a filter matched to m-sequence of rank 8 (**a**) and Kasami code of rank 8 (**b**); B = 1 kHz.

At the receiver side (Figure 1b), input signal y(t) is passed through the passband quadrature demodulator. Next, the complex-value digital signal is passed through the matched filter with complex-value coefficients $m_{c}\left(t\right)=m\left(t\right)+j\hat{m}\left(t\right)$, the real part of which corresponds to the PN sequence m(t) used in the transmitter, and the imaginary part $\hat{m}\left(t\right)$ is equal to the Hilbert transform of m(t). The output of the matched filter r(t) represents the cross-correlation function of signal y(t) and the sequence $m_{c}\left(t\right)$. It is passed to the input of the detection algorithm. Figure 2 shows real parts of exemplary signals r(t). They are divided into segments, each of length $N_{s}$, which correspond to modulation symbols.

The algorithm used for making the decision on the information represented by a single modulation symbol is as follows. The minimum $A_{min}$ and maximum $A_{max}$ values are determined for the real part of each segment, and their absolute values are compared with each other. If the maximum value $A_{max}$ is greater than the absolute value of $A_{min}$, then the modulation symbol represents a “1” bit, otherwise, a “0” bit. The rule for making the decision on received data $d_{r}$ can be expressed as

(1) $$d_{r}\left[n\right]=\left\{\begin{array}{cc} 1 & \mathrm{if}A_{max}^{n}>\left|A_{min}^{n}\right|\\ 0 & \mathrm{if}A_{max}^{n}\leq \left|A_{min}^{n}\right| \end{array}\right.$$

where $A_{max}^{n}=\max\left\{r^{n}\left(t\right)\right\}$, $A_{min}^{n}=\min\left\{r^{n}\left(t\right)\right\}$, and *n* is the number of the modulation symbol.

## 3. Simulation Tests Using Measurement Pool Simulator

The reliability of a UAC system using different DSSS signals was tested using a simulator of an underwater channel developed in the Department of Sonar Systems, Gdańsk University of Technology (Gdańsk Tech). The simulator, working in the MATLAB software environment, models the propagation conditions in the measurement pool of the Gdańsk Tech. It is based on the virtual source method. Based on input data such as the dimensions of the pool, the position of the transmitting and receiving transducers, the reflection coefficients from the pool walls (assumed to be equal to 0.7) and the water surface (equal to −1), and the number of virtual sources, the channel impulse responses between the transducers are calculated.

**Figure 3:**
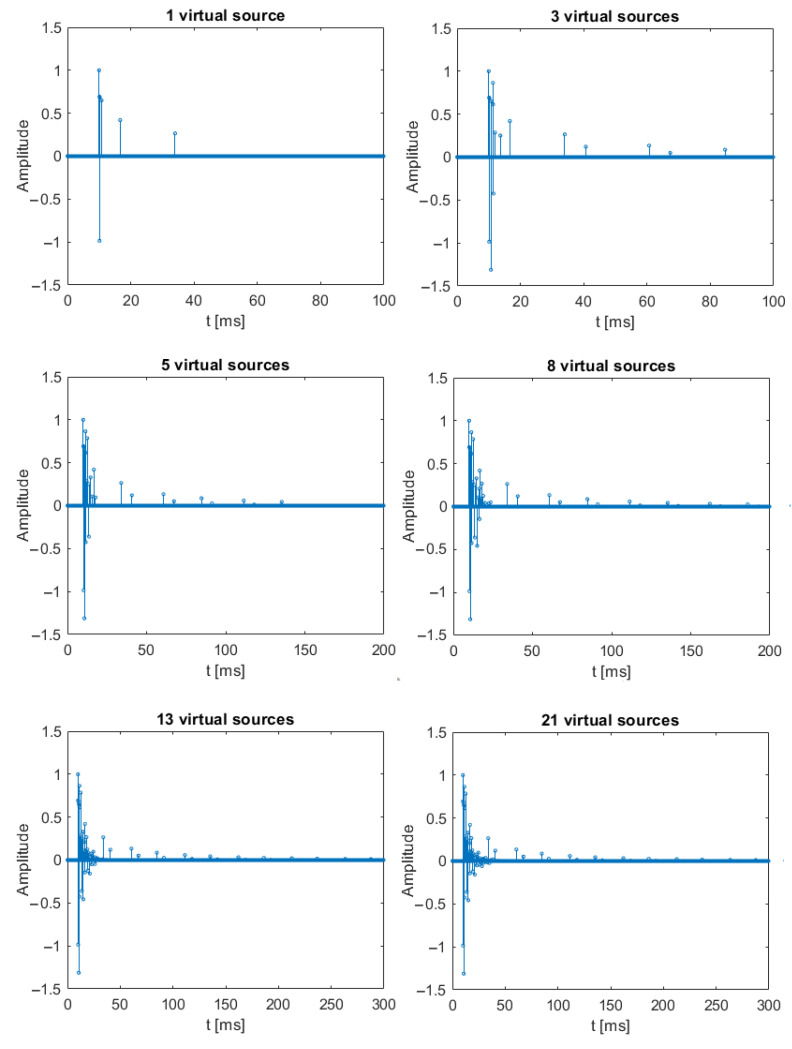
Impulse responses used during the simulation tests.

DSSS signals transmission was simulated using six impulse responses generated for 1, 3, 5, 8, 13, and 21 virtual sources. The pool’s size was assumed to be 40 m long, 4 m wide, and 3 m deep. It was assumed that the transmitting and receiving transducers are immersed to a depth of 1.5 m. The distance of the transmitting transducer from the pool’s edge was 10 m from the short edge and 1.5 m from the long edge. For the receiving transducer, the distances were, respectively, 15 m and 1.5 m. The impulse responses for each case of the number of virtual sources are shown in Figure 3.

**Figure 4:**
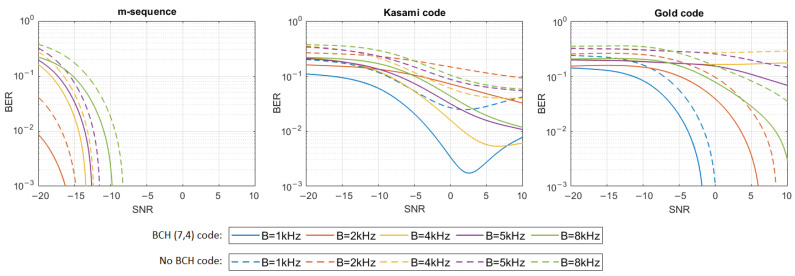
BER as a function of SNR for transmission in an UAC channel simulated using impulse response generated by 1 virtual source.

**Figure 5:**
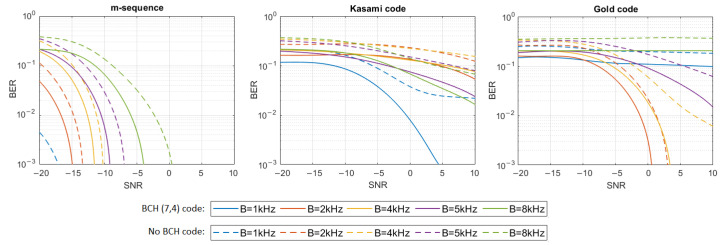
BER as a function of SNR for transmission in an UAC channel simulated using impulse response generated by 3 virtual sources.

**Figure 6:**
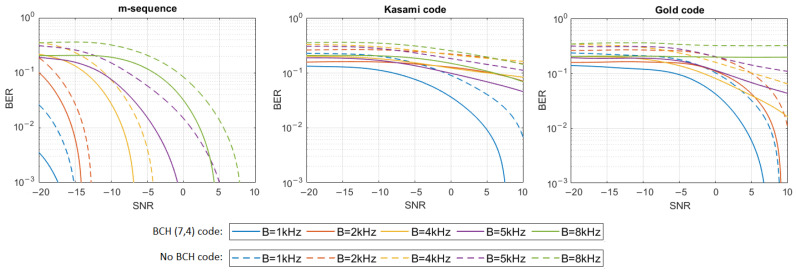
BER as a function of SNR for transmission in an UAC channel simulated using impulse response generated by 5 virtual sources.

**Figure 7:**
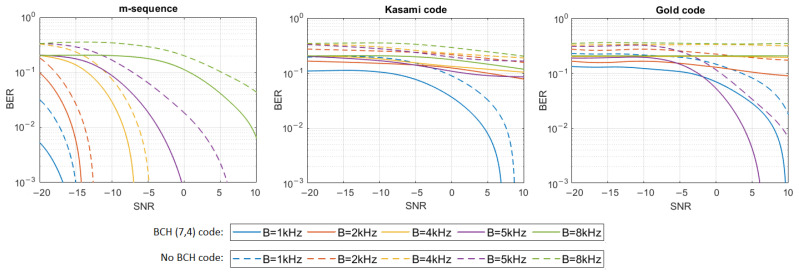
BER as a function of SNR for transmission in an UAC channel simulated using impulse response generated by 8 virtual sources.

**Figure 8:**
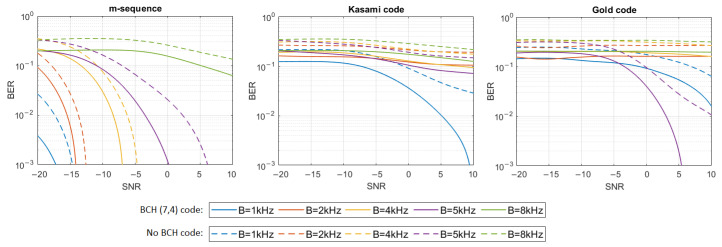
BER as a function of SNR for transmission in an UAC channel simulated using impulse response generated by 13 virtual sources.

**Figure 9:**
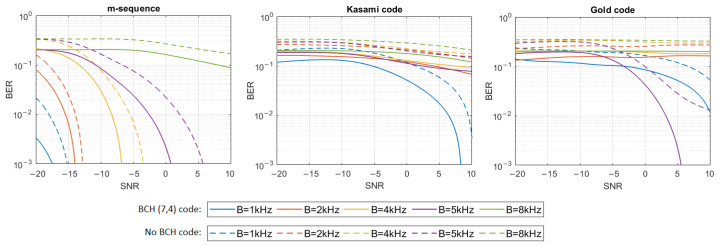
BER as a function of SNR for transmission in an UAC channel simulated using impulse response generated by 21 virtual sources.

The generated impulse responses were used as filters through which the DSSS signals were passed, and AWGN noise was added to the filter output:

(2) $$y\left[n\right]=\sum_{k=0}^{K-1}h[k]s[n-k]+w\left[n\right]$$

where h[n] represents the impulse response samples, s[n] and y[n] are digital representations of signals—transmitted s(t) and received y(t)—and w[n] are the samples of Additive White Gaussian Noise (AWGN). The simulation tests were performed in 16 different noise levels so that the Signal-to-Noise Ratio (SNR) varied from −20 dB to 10 dB. For each signal type, defined by the PN sequence and bandwidth used, and for each SNR, 20 tests were performed using different information bit strings, and then the average BER for all 20 tests was calculated. The results have shown that only m-sequences can make the transmission with a bit error rate lower than 0.001 in multipath propagation conditions possible. Therefore, additional simulation tests were carried out using Bose–Chaudhuri–Hocquenghem (BCH) Error Correction Coding (ECC) of the information carried by DSSS signals to check if encoding will significantly improve BER in the case of Gold codes and Kasami codes. BCH (7.4) code was used, which generates a 7-bit message to be transmitted for every 4 bits of information [18]. Figure 4, Figure 5, Figure 6, Figure 7, Figure 8 and Figure 9 show the obtained bit error rates as SNR functions for uncoded and BCH-coded signals. The curves represent polynomial trend lines of the results. For each transmitted signal, defined by the spreading sequence and the bandwidth used, minimum SNR values were determined for which transmission was obtained with reliability defined by a BER lower than 0.01 and lower than 0.001. The results for BCH-coded signals are shown in Table 2.

In the case of the impulse response generated using a single virtual source, transmission by m-sequences-based signals is possible with an error rate lower than 0.001 for each signal bandwidth. In the case of BCH-coded signals with a bandwidth equal to 1 kHz or 2 kHz, communication is possible even when the SNR is as low as −20 dB; however, for the 8 kHz bandwidth, the SNR should be not less than −10 dB. In the case of Kasami codes, transmission with a BER less than 0.001 is impossible, but BER less than 0.01 can be achieved in a bandwidth of 1 kHz if the SNR is greater than −6 dB. Using Gold codes, a BER less than 0.001 is possible for a bandwidth equal to 1 kHz when the SNR is no less than −2 dB, and for a bandwidth equal to 2 kHz, when the SNR is no less than 6 dB. In the 8 kHz bandwidth, BER less than 0.01 is possible to obtain when SNR is not less than 8 dB. In 4 kHz and 5 kHz bandwidths, the assumed BER is not achieved, even with the use of BCH codes.

In the UAC channel simulated with three or five virtual sources, the BCH-coded transmission was achieved with the assumed reliability using m-sequences in each frequency bandwidth. As the bandwidth increases, so does the required SNR. In the case of three virtual sources using Kasami codes, transmission with a BER < 0.01 is possible when the SNR is no less than −2 dB for the 1 kHz bandwidth. In the case of higher bandwidth, reliable transmission is not possible. Using the Gold codes, it is impossible to obtain a coded transmission with a BER < 0.001 or BER < 0.01 for the 1 kHz, 5 kHz, and 8 kHz bandwidths. Such a transmission (BER < 0.001) is possible for the 2 kHz and 4 kHz bandwidth when SNR is no less than 0 dB and 8 dB, respectively. In the case of five virtual sources using Kasami codes, transmission with a BER < 0.01 is possible only when the SNR is no less than 2 dB and only for the 1 kHz bandwidth. Using the Gold codes, transmission with a BER less than 0.001 can be achieved for the 1 kHz bandwidth and with a BER less than 0.01—for the 2 kHz bandwidth. In both cases SNR not less than 8 dB is required.

In the case of the most complex impulse responses, based on 13 and 21 virtual sources, reliable transmission in the 8 kHz bandwidth is not possible regardless of the spreading sequence used, even if it is BCH coded. In the remaining bands, such coded transmission with BER less than 0.001 is successful using m-sequence in conditions with a negative SNR (only for 5 kHz bandwidth positive SNR is required). For Kasami codes, the transmission is successful only in the 1 kHz bandwidth with a positive SNR, and for Gold codes, for the 5 kHz bandwidth when SNR is not less than 4 dB. In the case of 21 virtual sources, transmission with BER < 0.01 was achieved for 1 kHz bandwidth and SNR equal to 10 dB.

The simulation experiment has shown that, regardless of the multipath propagation conditions (the number of virtual sources), the use of m-sequences produces a much lower BER than the use of Gold codes or Kasami codes. BER less than 0.001 can be achieved for very low SNR for signals transmitted in the bandwidth of 1 kHz or 2 kHz. As the bandwidth of the signal increases, the resulting BER also increases. It is clearly seen that, in the case of Kasami codes and Gold codes for some values of the bandwidth, it is not possible to improve BER to the value lower than 0.01 using BCH (7,4) codes. If the number of virtual sources is greater than 3, this problem appears almost for every bandwidth value except 1 kHz.

## 4. Simulation Tests Using Watermark Replay Channel Simulator

The additional simulation tests of DSSS transmission were performed using the Watermark simulator. This is a freely available benchmark for physical-layer schemes for underwater acoustic communications. Its core is a replay channel simulator driven by at-sea measurements of the time-varying impulse response. For testing DSSS signals, all five communication channels available at Watermark were used, represented by impulse responses measured in Norway-Oslofjord (NOF1), Norway-Continental Shelf (NCS1), Brest Commercial Harbor (BCH1), and in shallow water off the western side of Kauai, HI, USA (KAU1 and KAU2). NOF1 and NCS1 impulse responses were recorded between a stationary source and a stationary single-hydrophone receiver. The BCH1 channel was measured with a source and a four-element array lowered into the water column from two docks. KAU1 and KAU2 impulse responses were recorded between a towed source and a vertically suspended array with 16 receivers. The main difference between KAU2 with KAU1 is the larger range, which attenuates delayed arrival clusters more rapidly [19]. The basic Watermark channels’ parameters and sounding conditions are shown in Table 3.

During the simulation tests, the DSSS signals of parameters shown in Table 1 were transmitted. Similarly, as in case tests performed using the measurement pool simulator, three types of spreading sequence were used, all of rank 8. Depending on the Watermark channel type, different carrier frequencies and maximum possible bandwidths were used to fit the DSSS signal to the frequency band in which the impulse responses of the given channel have been measured. In the case of the NOF1 and NCS channels, the maximum bandwidth was equal to 8 kHz, and carrier frequency was equal to 14 kHz. For the BCH1 channel these values were equal to 5 kHz and 35 kHz, respectively. In the case of KAU channels, the maximum bandwidth was equal to 4 kHz, and the carrier frequency was equal to 6 kHz.

Based on the BER values obtained, the coding rate of BCH error correction coding necessary to obtain the reliability expressed as BER less than 0.01 and 0.001 was estimated. Than the possibility of obtaining such an error rate was confirmed in additional simulations using Watermark.

Table 4, Table 5, Table 6, Table 7 and Table 8 present the results of all simulation tests performed using the Watermark software. Each table shows the results for one replay channel. The second and the third columns present BER values and transmission rates achieved without BCH coding. In the following pairs of columns, the BCH code rates required to obtain BER < 0.01 and BER < 0.001 and the corresponding transmission rates are presented. The code rate is defined as $C_{r}=L_{i}/L_{msg}$, where $L_{i}$ is a number information bits and $L_{msg}=2^{m}-1$ is message length, m∈{3,4,5,6,7,8,9,10,11,12,13}.

Similarly, as in the tests with the measurement pool simulator, the best BER results were obtained for the DSSS signal bandwidth equal to 1 kHz. In this case, the use of each of the spreading sequences makes it possible to achieve a BER less than 0.001 on each tested channel. The possible transmission rate corresponding to such bandwidth is 3.92 bps (Table 1).

In the bandwidth equal to 2 kHz, a BER less than 0.001 was obtained for the NCS1 and BCH1 channels, regardless of the spreading sequence. In the case of the NOF1 channel, this result was possible to obtain using only the m-sequence. In other cases, the BER was no higher than 0.05. Thus it can be improved to less than 0.001 using the BCH coding of maximum code rate equal to 6/31 = 0.1935, reducing the transmission rate from 7.84 bps to 1.52 bps in the KAU1 channel.

For a bandwidth equal to or greater than 4 kHz, a significant deterioration in the obtained bit error rate was observed. The BER value of rank 0.3 cannot be improved to a level of less than 0.01. This would require BCH code with a code rate of rank 12/2047 = 0.0059 or 13/4095 = 0.0032, which would reduce the bitrate to less than 0.1 bps, which, in turn, would make such a UAC system useless.

Among all the tests carried out in the Watermark simulator, the highest transmission rate (equal to 7.84 bps) with reliability expressed as BER less 0.001 was obtained in the 2 kHz bandwidth using the m-sequence in the NOF1 channel and with any 8-order sequence in the NCS1 and BCH1 channels.

## 5. Experimental Tests

**Figure 10:**
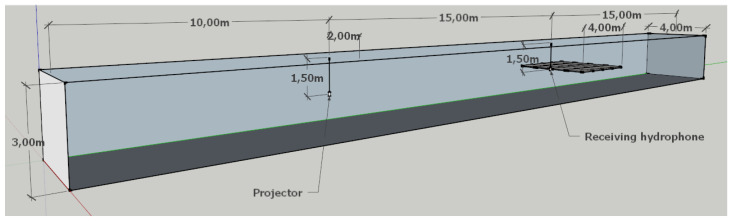
The dimensions of the model pool and the location of the transmitting (projector) and receiving transducers.

Experimental tests of DSSS communication were carried out in the model pool of the Gdańsk Tech. The pool is 40 m long, 4 m wide, and 3 m deep. The location of the transmitting and receiving hydrophone is shown in Figure 10 and corresponds to the system configuration assumed during the simulation tests using measurement pool simulator. Transducers were immersed at a depth of 1.5 m.

Both the transmitter and receiver of the UAC system’s laboratory model use laptop computers with the Matlab environment for digital signal generation and analysis. Laptop computers communicate with underwater HTL-10 telephones from Sonel Sp. z o.o., developed for the needs of the Polish Navy. It performs the generation of the communication signal and an analysis of the received signals using digital signal processors by Texas Instruments: a 16-bit TMS320VC5416 fixed-point processor and a TMS320C6713B (DSP) 32-bit floating-point processor. It contains multichannel analog-to-digital converters with a 16-bit resolution and a maximum sampling frequency of 250 kHz. The source of the sampling frequency is an AD9834 direct digital synthesis circuit from Analog Devices. The underwater telephone works with an NI-USB6363 external recording and generating device from National Instruments. The HTL-10 devices pass the analog signal to a hydroacoustic transducer and receive the signal from a receiving transducer. Both the transmitting and receiving transducers are omnidirectional transducers with a resonant frequency of 34 kHz.

Using such a laboratory model of the UAC system, DSSS communication tests were carried out, preceded by the measurement of the UAC channel transmission parameters, determining the degree of multipath propagation.

### 5.1. Transmission Parameters of UAC Channel

The multipath propagation phenomenon’s intensity can be expressed with two basic transmission parameters of the communication channel, namely, the delay spread and the coherence bandwidth. In order to estimate these parameters, the Time-Varying Impulse Response (TVIR) was measured by the correlation method with the use of a Pseudo-Random Binary Sequence (PRBS) signal, based on an m-sequence of rank 8. The carrier frequency $f_{c}$ and the sampling frequency $f_{s}$ in each case were equal to 30 kHz and 200 kHz, respectively. The impulse response was measured using a PRBS signal of 5 different bandwidths, corresponding to the communication signal’s bandwidths.

For measured bandpass impulse responses, its complex baseband equivalent h(t,τ) was calculated, defined in a window of observation time *t*, and delay τ. A maximum value of delay t is a duration of a single realization of TVIR. It is equal to a single-probe sequence duration, that is, $T_{s}=\frac{R\cdot L}{f_{s}}=102.3\;\mathrm{ms}$, where $L=2^{8}-1$ denotes the number of m-sequence bits and *R* denotes the upsampling factor, which is equal to $\frac{f_{s}}{B}$, where *B* is the bandwidth of the PRBS signal. The probe sequence was repeated numerous times, which allowed the capture of up to 48 realizations of the impulse response. Thus, h(t,τ) is a discrete-time function defined in the τ domain with a resolution equal to $1/f_{s}$, and in the *t* domain with a resolution equal to probe signal duration $T_{s}$.

TVIR is a basis for calculating the Time-Varying Transfer Function (TVTF) H(t,f) with the use of Fourier transform. The autocorrelation function of TVIF is called the Space-Time-Frequency Correlation Function (STFCF) $R_{h}\left(\Delta t,\Delta f\right)$. It is a basis for calculating the 2-dimensional Scattering Function (SF) in the manner described by Equation (3):

(3) $$S\left(\nu ,\tau \right)=\int_{\Delta t}\int_{\Delta f}R_{h}\left(\Delta t,\Delta f\right)e^{-j2\pi (\nu \Delta t-\tau \Delta f)}d\Delta td\Delta f$$

where Δt and Δf are time and frequency differences, respectively, ν is the Doppler spread, τ is the delay, and $R_{h}\left(\Delta t,\Delta f\right)$ is the STFCF of the channel. The integral of S(ν,t) over ν gives the Power Delay Profile (PDP) P(τ). Based on PDP, the delay spread is obtained as a maximum delay spread $\tau_{M}$ measured at a threshold level of 0.1 of a maximum value of P(τ), and as root–mean–square (rms) value $\tau_{rms}$:

(4) $$\tau_{rms}=\sqrt{\bar{\tau^{2}}-\tau_{m}^{2}}$$

where:

$$\bar{\tau^{2}}=\frac{\sum_{k}P\left(\tau_{k}\right)\tau_{k}^{2}}{\sum_{k}P(\tau_{k})},\;\tau_{m}^{2}=\frac{\sum_{k}P\left(\tau_{k}\right)\tau_{k}}{\sum_{k}P(\tau_{k})}$$

Averaging R(Δt,Δf) over the Δt domain gives the Space–Frequency Correlation Function (SFCF) R(Δf). The coherence bandwidth $B_{c}$ is calculated as the width of R(Δf) at a threshold level $T_{r}$ equal to 0.5 and 0.7 [20].

**Figure 11:**
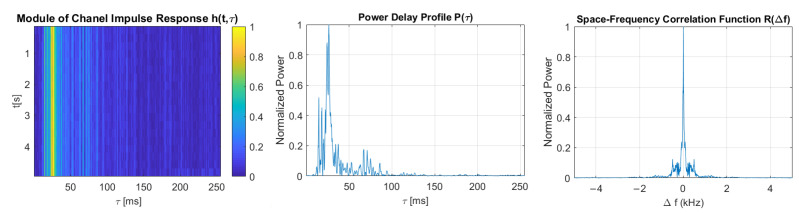
The module of time-varying impulse response, measured using PRBS of a bandwidth equal to 1 kHz, and estimated power delay profile and space-frequency correlation function.

**Figure 12:**
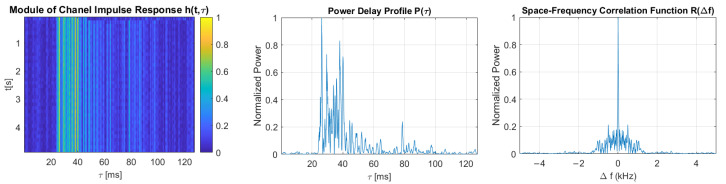
The module of time-varying impulse response, measured using PRBS of a bandwidth equal to 2 kHz, and estimated power delay profile and space-frequency correlation function.

**Figure 13:**
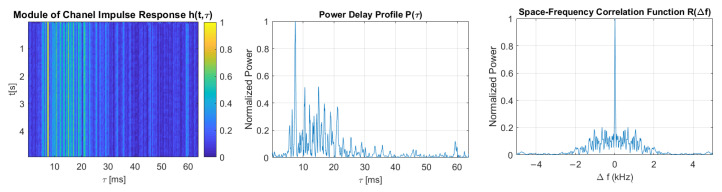
The module of time-varying impulse response, measured using PRBS of a bandwidth equal to 4 kHz, and estimated power delay profile and space-frequency correlation function.

**Figure 14:**
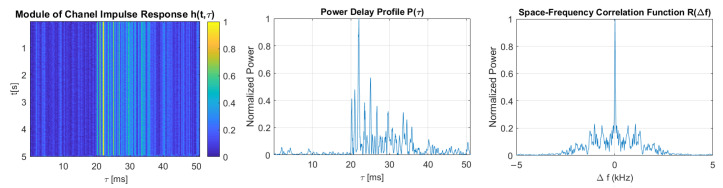
The module of time-varying impulse response, measured using PRBS of a bandwidth equal to 5 kHz, and estimated power delay profile and space-frequency correlation function.

**Figure 15:**
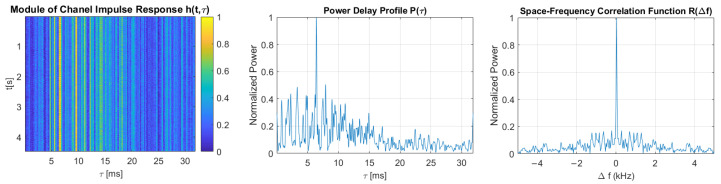
The module of time-varying impulse response, measured using PRBS of a bandwidth equal to 8 kHz, and estimated power delay profile and space-frequency correlation function.

Figure 11, Figure 12, Figure 13, Figure 14 and Figure 15 show the modules of TVIRs, PDPs, and SFCFs estimated based on measurements performed by PRBS signals of different bandwidths. It can be clearly seen in the plots of modules of TVIRs and PDPs that the relative intensity of multipath propagation increases with the increased signal bandwidth. The shape of the SFCF also changes as the probe signal bandwidth increases. The transmission parameters of a measured UAC channel are shown in Table 9. The values vary depending on the bandwidth of the measurement signal. The maximum delay spread, estimated on the basis of TVIR measured with a sequence of duration 255 ms, is equal to about 62 ms, and this is almost twice as long as the one estimated from the measurement with a signal with a bandwidth of 8 kHz. The rms values of the delay spread differ even more. The value estimated in the case of the 1 kHz measurement signal is four times larger than the one estimated based on the results of measurements performed at a bandwidth of 8 kHz. The values of the coherence bandwidth estimated at the level of 0.5 of the maximum value of SFCF are between 47 Hz and 79 Hz, while the range of $B_{C}$ values calculated at the level of 0.9 is much larger, from 15.68 Hz to 62.73 Hz.

For each DSSS signal described in Table 1, the modulation symbol duration exceeds both the maximum and rms values of the delay spread, regardless of the probe signal’s bandwidth. Thus, it meets the basic condition of avoiding ISI in the time domain, which is that the duration of the transmitted symbol should be longer than the delay spread of the channel [4]. None of the transmitted signals meet the condition for avoiding the frequency-selective fading of the signal spectrum, which is that the signal bandwidth should be smaller than the channel’s coherence bandwidth. However, it is difficult to meet such a condition in a channel with strong multipath propagation if the spread spectrum modulation technique is used.

### 5.2. Communication Tests

The communication tests in the model pool were performed using DSSS signals constructed of m-sequences, Kasami codes, and Gold codes. The parameters of the transmitted signals are presented in Table 1. For each signal parameter’s configuration, 20 transmission tests were performed. During each test, a single transmission frame had a duration of 6 s and was constructed of a variable number of symbols, depending on the signal bandwidth (Table 1). The set of information bits sent in each transmission frame was different, generated as a pseudorandom sequence with uniform distribution. During each communication test, regardless of the bandwidth of the transmitted signal, the received signal-to-noise ratio was in the range of 15–17 dB.

After performing the measurements and analysis of the obtained results, additional simulation tests were carried out to estimate how the obtained reliability can be increased using ECC coding. For this, sequences of correctly and incorrectly received bits were used, achieved during the processing of the recorded DSSS signals. The same BCH codes were used, as in the case of simulation tests performed in the Watermark simulator.

Table 10 shows the BER results averaged over 20 transmission tests for each type of communication signal, determined by the spreading sequence and the bandwidth. Similar to the tests with the measurement pool simulator and Watermark software, the best BER results were obtained for the DSSS signal bandwidth equal to 1 kHz. In this case, the use of each of the spreading sequences makes it possible to achieve a BER less than 0.001.

In the case of other bandwidth values, the best results were obtained using the m-sequence as the spreading sequence. The BER obtained for Kasami codes and Gold was several times higher than in the case of m-sequences. During the transmission of signals of bandwidth equal to 2 kHz, m-sequence could obtain a BER less than 0.001, while for Kasami codes the BER was equal to 0.06, and for Gold codes the BER was equal to 0.18. Using ECC encoding, it is possible to achieve an error rate of less than 0.01, with a code rate equal to 5/15 = 0.3333 in the case of transmission with Kasami codes. The transmission rate is then reduced from 7.84 bps to 2.61 bps. Moreover, a BER less than 0.001 can be achieved using a BCH code rate equal to 6/31 = 0.1935, which means a reduction in the transmission rate to 1.62 bps. In the case of transmission with Gold codes, achieving a BER lower than 0.01 would reduce the transmission rate to a value lower than 1 bps.

In the bandwidth equal to 4 kHz, it is possible to transmit data with a rate equal to 5.23 bps (m-sequences) or 3.04 bps (Gold codes), with a BER less than 0.01. A BER less than 0.001 can be achieved in the case of transmission with m-sequences with a rate equal to 3.04 bps.

During transmission in a bandwidth equal to 5 kHz, it is possible to obtain a BER of less than 0.01 using the m-sequences and BCH coding of code rate equal to 7/63 = 0.1111. The ECC coding reduces the transmission rate from 19.61 bps to 2.18 bps.

In the bandwidth equal to 8 kHz, the highest reliable transmission rates using m-sequences were obtained, that is, 10.46 bps with a BER less than 0.01 and 6.07 bps with a BER less than 0.001.

## 6. Conclusions

The idea of the spreading spectrum technique is to widen the signal bandwidth in order to obtain the desired transmission rate and reliability at a given noise level. However, as demonstrated by the simulation and experimental tests, in conditions of strong multipath propagation, extending the signal bandwidth deteriorates data transmission reliability. This is because of the frequency-selective fading observed in the spectrum of the signal, which undergoes numerous reflections. The simulation tests performed with the use of a measurement pool simulator have shown that in the case of a small number of signal reflections reaching the receiver (channel model with 1 virtual source), it is possible to obtain reliable data transmission at any transmission rate resulting from the bandwidth. Increasing the number of reflections of the signal has no effect on the results achieved with m-sequence in the 1 kHz and 2 kHz bandwidths, but further increasing the bandwidth increases the required SNR.

In each of the simulation and experimental tests of the transmission of signals in a bandwidth equal to 1 kHz, the reliability described by the BER less than 0.001 was achieved without the need to use ECC encoding. However, the relatively narrow bandwidth limits the transmission rate to 3.92 bps. In cases where bandwidth is equal to 2 kHz, slightly worse BER values were obtained; however, it is possible to reach a BER value of less than 0.01 or 0.001 when using reasonably redundant ECC encoding.

During the simulation tests using signals of bandwidth equal to or greater than 4 kHz, significantly worse reliability results were obtained than for bandwidths equal to 1 kHz and 2 kHz. In the case of BER obtained in the Watermark simulator, this value cannot be improved to the level of 0.01 while maintaining a useful data transmission rate.

During the experimental tests in the measuring pool, the BER achieved in bandwidth equal to 8 kHz was much lower than in simulation tests. Therefore, future analysis of the adequacy of the simulation UAC channel models for real conditions in the measuring pool is necessary when the UAC system operates within s wide band. Moreover, in strong multipath propagation conditions, it is difficult to measure the transmission parameters of the underwater channel. The probe signals used are naturally wideband because the bandwidth is only an order of magnitude smaller than the value of the carrier frequency. Therefore, probe signals with a bandwidth of 1 kHz and 8 kHz make it possible to obtain significantly different estimates of the channel transmission parameters. This may also make it difficult to design the DSSS data transmission parameters, the physical layer of which should be adjusted to the channel’s transmission properties.

In all the simulation and experimental tests performed, using m-sequences allowed for better DSSS transmission reliability than the use of Gold and Kasami codes as spreading sequences.

## Figures and Tables

**Table 1:** DSSS signals parameters.

Bandwidth *B*	Upsampling Factor *R*	Symbol Length $N_{s}$	Symbol Duration $T_{s}$	Symbols per Frame *K*	Data Transmission Rate
1 kHz	200	51,000	255.00 ms	23	3.92 bps
2 kHz	100	25,500	127.50 ms	47	7.84 bps
4 kHz	50	12,750	63.75 ms	94	15.69 bps
5 kHz	40	10,200	51.00 ms	117	19.61 bps
8 kHz	25	6375	31.88 ms	188	31.37 bps

**Table 2:** The minimum SNR values for which BER < 0.01 (A) and BER < 0.001 (B) were achieved during the simulated transmission of signals coded with BCH(7,4) code.

Virtual Sources	Spreading Sequence	*B* = 1 kHz		*B* = 2 kHz		*B* = 4 kHz		*B* = 5 kHz		*B* = 8 kHz	
		A	B	A	B	A	B	A	B	A	B
1	m-sequence	−20 dB	−20 dB	−18 dB	−18 dB	−14 dB	−14 dB	−14 dB	−12 dB	−12 dB	−10 dB
1	Kasami code	−6 dB	—	—	—	—	—	—	—	—	—
1	Gold code	−4 dB	−2 dB	4 dB	6 dB	—	—	—	—	8 dB	—
3	m-sequence	−20 dB	−20 dB	−18 dB	−14 dB	−14 dB	−12 dB	−10 dB	−8 dB	−4 dB	−2 dB
3	Kasami code	−2 dB	—	—	—	—	—	—	—	—	—
3	Gold code	—	—	2 dB	0 dB	2 dB	8 dB	—	—	—	—
5	m-sequence	−20 dB	−18 dB	−16 dB	−14 dB	−8 dB	−6 dB	−2 dB	2 dB	2 dB	6 dB
5	Kasami code	2 dB	—	—	—	—	—	—	—	—	—
5	Gold code	4 dB	8 dB	8 dB	—	—	—	—	—	—	—
8	m-sequence	−20 dB	−18 dB	−16 dB	−14 dB	−8 dB	−6 dB	−2 dB	2 dB	10 dB	−−−
8	Kasami code	2 dB	10 dB	—	—	—	—	—	—	—	—
8	Gold code	8 dB	—	—	—	—	—	4 dB	10 dB	—	—
13	m-sequence	−20 dB	−18 dB	−16 dB	−14 dB	−8 dB	−6 dB	−4 dB	2 dB	—	—
13	Kasami code	4 dB	—	—	—	—	—	—	—	—	—
13	Gold code	—	—	—	—	—	—	4 dB	—	—	—
21	m-sequence	−20 dB	−18 dB	−16 dB	−14 dB	−8 dB	−4 dB	−2 dB	2 dB	—	—
21	Kasami code	8 dB	10 dB	—	—	—	—	—	—	—	—
21	Gold code	10 dB	—	—	—	—	—	4 dB	—	—	—

**Table 3:** Watermark channels’ parameters and sounding conditions [19].

Channel	NOF1	NCS1	BCH1	KAU1	KAU2
Environment	Fjord	Shelf	Harbour	Shelf	Shelf
Range	750 m	540 m	800 m	1080 m	3160 m
Water depth	10 m	80 m	20 m	100 m	100 m
3 dB frequency band	10–18 kHz	10–18 kHz	32.5–37.5 kHz	4–8 kHz	4–8 kHz
Delay coverage	128 ms	32 ms	102 ms	128 ms	128 ms
Doppler coverage	7.8 Hz	31.4 Hz	9.8 Hz	7.8 Hz	7.8 Hz
Type	SISO	SISO	SIMO	SIMO	SIMO

**Table 4:** Results of simulation tests using Watermark software; NOF1 channel.

Spreading Sequence	BER No ECC	Transmission Rate No ECC	BCH Code Rate BER < 0.01	Transmission Rate BER < 0.01	BCH Code Rate BER < 0.01	Transmission Rate BER < 0.01
**Bandwidth = 1 kHz**						
m-sequence	<0.001	3.92 bps	—	3.92 bps	—	3.92 bps
Kasami code	<0.001	3.92 bps	—	3.92 bps	—	3.92 bps
Gold code	<0.001	3.92 bps	—	3.92 bps	—	3.92 bps
**Bandwidth = 2 kHz**						
m-sequence	<0.001	7.84 bps	—	7.84 bps	—	7.84 bps
Kasami code	0.01	7.84 bps	4/7	4.48 bps	5/15	2.61 bps
Gold code	0.05	7.84 bps	5/15	2.61 bps	6/31	1.52 bps
**Bandwidth = 4 kHz**						
m-sequence	0.33	15.69 bps	12/2047	0.09 bps	>13/4095	<0.05 bps
Kasami code	0.26	15.69 bps	11/1023	0.17 bps	>12/2047	0.09 bps
Gold code	0.34	15.69 bps	12/2047	0.09 bps	>13/4095	<0.05 bps
**Bandwidth = 5 kHz**						
m-sequence	0.36	19.61 bps	12/2047	0.11 bps	>13/4095	<0.06 bps
Kasami code	0.36	19.61 bps	12/2047	0.11 bps	>13/4095	<0.06 bps
Gold code	0.33	19.61 bps	12/2047	0.11 bps	>13/4095	<0.06 bps
**Bandwidth = 8 kHz**						
m-sequence	0.38	31.37 bps	>13/4095	<0.1 bps	>13/4095	<0.1 bps
Kasami code	0.40	31.37 bps	>13/4095	<0.1 bps	>13/4095	<0.1 bps
Gold code	0.38	31.37 bps	>13/4095	<0.1 bps	>13/4095	<0.1 bps

**Table 5:** Results of simulation tests using Watermark software; NCS1 channel.

Spreading Sequence	BER No ECC	Transmission Rate No ECC	BCH Code Rate BER < 0.01	Transmission Rate BER < 0.01	BCH Code Rate BER < 0.01	Transmission Rate BER < 0.01
**Bandwidth = 1 kHz**						
m-sequence	<0.001	3.92 bps	—	3.92 bps	—	3.92 bps
Kasami code	<0.001	3.92 bps	—	3.92 bps	—	3.92 bps
Gold code	<0.001	3.92 bps	—	3.92 bps	—	3.92 bps
**Bandwidth = 2 kHz**						
m-sequence	<0.001	7.84 bps	—	7.84 bps	—	7.84 bps
Kasami code	<0.001	7.84 bps	—	7.84 bps	—	7.84 bps
Gold code	<0.001	7.84 bps	—	7.84 bps	—	7.84 bps
**Bandwidth = 4 kHz**						
m-sequence	0.35	15.69 bps	12/2047	0.09 bps	>13/4095	<0.05 bps
Kasami code	0.22	15.69 bps	7/63	1.74 bps	>10/511	0.31 bps
Gold code	0.35	15.69 bps	12/2047	0.09 bps	>13/4095	<0.05 bps
**Bandwidth = 5 kHz**						
m-sequence	0.37	19.61 bps	12/2047	0.11 bps	>13/4095	<0.06 bps
Kasami code	0.33	19.61 bps	12/2047	0.11 bps	>13/4095	<0.06 bps
Gold code	0.37	19.61 bps	12/2047	0.11 bps	>13/4095	<0.06 bps
**Bandwidth = 8 kHz**						
m-sequence	0.40	31.37 bps	>13/4095	<0.1 bps	>13/4095	<0.1 bps
Kasami code	0.38	31.37 bps	>13/4095	<0.1 bps	>13/4095	<0.1 bps
Gold code	0.40	31.37 bps	>13/4095	<0.1 bps	>13/4095	<0.1 bps

**Table 6:** Results of simulation tests using Watermark software; BCH1 channel.

Spreading Sequence	BER No ECC	Transmission Rate No ECC	BCH Code Rate BER < 0.01	Transmission Rate BER < 0.01	BCH Code Rate BER < 0.01	Transmission Rate BER < 0.01
**Bandwidth = 1 kHz**						
m-sequence	<0.001	3.92 bps	—	3.92 bps	—	3.92 bps
Kasami code	<0.001	3.92 bps	—	3.92 bps	—	3.92 bps
Gold code	<0.001	3.92 bps	—	3.92 bps	—	3.92 bps
**Bandwidth = 2 kHz**						
m-sequence	<0.001	7.84 bps	—	7.84 bps	—	7.84 bps
Kasami code	<0.001	7.84 bps	—	7.84 bps	—	7.84 bps
Gold code	<0.001	7.84 bps	—	7.84 bps	—	7.84 bps
**Bandwidth = 4 kHz**						
m-sequence	0.35	15.69 bps	12/2047	0.09 bps	>13/4095	<0.05 bps
Kasami code	0.23	15.69 bps	7/63	1.74 bps	10/511	0.31 bps
Gold code	0.35	15.69 bps	12/2047	0.09 bps	>13/4095	<0.05 bps
**Bandwidth = 5 kHz**						
m-sequence	0.37	19.61 bps	>13/4095	<0.06 bps	>13/4095	<0.06 bps
Kasami code	0.33	19.61 bps	12/2047	0.11 bps	>13/4095	<0.06 bps
Gold code	0.37	19.61 bps	>13/4095	<0.06 bps	>13/4095	<0.06 bps

**Table 7:** Results of simulation tests using Watermark software; KAU1 channel.

Spreading Sequence	BER No ECC	Transmission Rate No ECC	BCH Code Rate BER < 0.01	Transmission Rate BER < 0.01	BCH Code Rate BER < 0.01	Transmission Rate BER < 0.01
**Bandwidth = 1 kHz**						
m-sequence	<0.001	3.92 bps	—	3.92 bps	—	3.92 bps
Kasami code	<0.001	3.92 bps	—	3.92 bps	—	3.92 bps
Gold code	<0.001	3.92 bps	—	3.92 bps	—	3.92 bps
**Bandwidth = 2 kHz**						
m-sequence	0.04	7.84 bps	5/15	2.61 bps	6/31	1.52 bps
Kasami code	0.01	7.84 bps	4/7	4.48 bps	5/15	2.61 bps
Gold code	0.02	7.84 bps	4/7	4.48 bps	5/15	2.61 bps
**Bandwidth = 4 kHz**						
m-sequence	0.35	15.69 bps	13/4095	0.05 bps	>13/4095	<0.05 bps
Kasami code	0.28	15.69 bps	12/20147	0.09 bps	>13/4095	<0.05 bps
Gold code	0.33	15.69 bps	13/4095	0.05 bps	>13/4095	<0.05 bps

**Table 8:** Results of simulation tests using Watermark software; KAU2 channel.

Spreading Sequence	BER No ECC	Transmission Rate No ECC	BCH Code Rate BER < 0.01	Transmission Rate BER < 0.01	BCH Code Rate BER < 0.01	Transmission Rate BER < 0.01
**Bandwidth = 1 kHz**						
m-sequence	<0.001	3.92 bps	—	3.92 bps	—	3.92 bps
Kasami code	<0.001	3.92 bps	—	3.92 bps	—	3.92 bps
Gold code	<0.001	3.92 bps	—	3.92 bps	—	3.92 bps
**Bandwidth = 2 kHz**						
m-sequence	0.007	7.84 bps	—	7.84 bps	5/7	4.48 bps
Kasami code	0.02	7.84 bps	4/7	4.48 bps	5/15	2.61 bps
Gold code	0.02	7.84 bps	4/7	4.48 bps	5/15	2.61 bps
**Bandwidth = 4 kHz**						
m-sequence	0.35	15.69 bps	13/4095	0.05 bps	>13/4095	<0.05 bps
Kasami code	0.26	15.69 bps	12/20147	0.09 bps	>13/4095	<0.05 bps
Gold code	0.35	15.69 bps	13/4095	0.05 bps	>13/4095	<0.05 bps

**Table 9:** Transmission parameters of UAC channel.

*B*	$T_{s}$	$\tau_{M}$	$\tau_{rms}$	$B_{c}$, $T_{R}=0.5$	$B_{c}$, $T_{R}=0.7$
1 kHz	255.00 ms	61.64 ms	37.54 ms	54.90 Hz	15.68 Hz
2 kHz	127.50 ms	62.36 ms	21.77 ms	47.05 Hz	15.68 Hz
4 kHz	63.75 ms	54.12 ms	13.54 ms	62.75 Hz	31.37 Hz
5 kHz	51.00 ms	20.23 ms	9.29 ms	78.43 Hz	39.22 Hz
8 kHz	31.88 ms	31.88 ms	8.15 ms	62.73 Hz	62.73 Hz

**Table 10:** Results of experimental tests.

Spreading Sequence	BER No ECC	Transmission Rate No ECC	BCH Code Rate BER < 0.01	Transmission Rate BER < 0.01	BCH Code Rate BER < 0.01	Transmission Rate BER < 0.01
**Bandwidth = 1 kHz**						
m-sequence	<0.001	3.92 bps	—	3.92 bps	—	3.92 bps
Kasami code	<0.001	3.92 bps	—	3.92 bps	—	3.92 bps
Gold code	<0.001	3.92 bps	—	3.92 bps	—	3.92 bps
**Bandwidth = 2 kHz**						
m-sequence	<0.001	7.84 bps	—	7.84 bps	—	7.84 bps
Kasami code	0.06	7.84 bps	5/15	2.61 bps	6/31	1.62 bps
Gold code	0.18	7.84 bps	7/63	0.87 bps	8/127	0.49 bps
**Bandwidth = 4 kHz**						
m-sequence	0.07	15.69 bps	5/15	5.23 bps	6/31	3.04 bps
Kasami code	0.33	15.69 bps	12/2047	0.09 bps	>13/4095	<0.05 bps
Gold code	0.17	15.69 bps	6/31	3.04 bps	8/127	0.99 bps
**Bandwidth = 5 kHz**						
m-sequence	0.20	19.61 bps	7/63	2.18 bps	11/1023	0.21 bps
Kasami code	0.28	19.61 bps	12/2047	0.11 bps	>13/4095	<0.06 bps
Gold code	0.25	19.61 bps	11/1023	0.21 bps	12/2047	0.11 bps
**Bandwidth = 8 kHz**						
m-sequence	0.08	31.37 bps	5/15	10.46 bps	6/31	6.07 bps
Kasami code	0.31	31.37 bps	12/2047	0.18 bps	>13/4095	<0.1 bps
Gold code	0.17	31.37 bps	6/31	6.07 bps	8/127	1.98 bps

## Data Availability

Not applicable.

## Abbreviations

The following abbreviations are used in this manuscript:

BER	Bit Error Rate
BCH	Bose-Chaudhuri-Hocquenghem
CDMA	Code-Division Multiple Access
ECC	Error Correction Coding
ISI	Inter-Symbol Interference
PDP	Power Delay Profile
PRBS	Pseudo-Random Binary Sequence
SF	Scattering Function
SFCF	Space-Frequency Correlation Function
SNR	Signal-to-Noise Ratio
STFCF	Space-Time-Frequency Correlation Function
TVIR	Time-Varying Impulse Responses
TVTF	Time-Varying Transfer Function
UAC	Underwater Acoustic Communications
